# Analysis of Hazard Rate of Municipalities in Slovakia in Terms of COVID-19

**DOI:** 10.3390/ijerph18179082

**Published:** 2021-08-28

**Authors:** František Petrovič, Katarína Vilinová, Radovan Hilbert

**Affiliations:** 1 Department of Ecology and Environmental Sciences, Faculty of Natural Sciences, Constantine the Philosopher University, 949 01 Nitra, Slovakia; fpetrovic@ukf.sk; 2 Department of Geography and Regional Development, Faculty of Natural Sciences, Constantine the Philosopher University, 949 01 Nitra, Slovakia; 3 Department of Fire Protection, Faculty of Wood Sciences and Technology, Technical University in Zvolen, YMS, a. s., 960 01 Trnava, Slovakia; radovan.hilbert@gmail.com

**Keywords:** COVID-19 pandemic, Slovakia, exposure, hazard

## Abstract

The coronavirus became a phenomenon in 2020, which is making an unwanted but wide space for the study of various scientific disciplines. The COVID-19 pandemic situation which has reached almost the whole civilized world by its consequences thus offers a unique possibility to analyze the graphic space and the human activities inside it. The aim of this study is to predict and identify the potential rate of threat on the example of COVID-19 in Slovakia through an established model. This model consisted of an assessment of the partial phenomena of exposure, vulnerability, and overall risk. The statistical data used to evaluate these phenomena concerned individual cities in Slovakia. These represent the smallest administrative unit. Indirect methods based on the point method were applied in the paper. The spreading and transfer of the disease was influenced much more by the exposure presented by traffic availability, especially, but also the concentration of inhabitants in the selected locations (shops, cemeteries, and others). In the results, our modeling confirmed the regions with the highest intensity, especially in the districts (Bratislava, Košice, Prešov, and Nitra). The selection of the data and method used in this study together with the results reached and presented may serve as an appropriate tool for the support of decision-making of other measures for the future.

## 1. Introduction

After the year 2000, new infectious diseases began to appear. These diseases are occurring on an increasing scale and frequency, for example, the Ebola virus disease, severe acute respiratory syndrome (SARS), avian influenza and pandemic influenza, respiratory syndrome at the Near East (MERS), and the recently discovered coronavirus disease 2019 (COVID-19). In addition to these mostly global-spread infectious diseases, it is necessary to also focus on the transfer of other infectious diseases such as influenza, viral angina, sheep pox, and measles. The true infectious diseases are characterized by the fact that the disease-carrying elements have adapted to life in the respiratory tract from where they are spread to the air when speaking, sneezing, and coughing, through the respiratory tract to penetrate other individuals. If this individual is close to the source of infection, it is possible to become infected by inhaling these elements from the air (the so-called droplet infection). This is how all the respiratory tract diseases are transferred, so this is a universal path for transfer of diseases from this group. At the same time, these create the most widespread infections due to their easy transfer [1]. The source of infection is the infected individual who by coughing and sneezing, and also by simply breathing, spreads small infected droplets of infection to the air. The individuals being located close to this infected person may thus become infected very easily. The carriers of the respiratory diseases transferred in the air are the cores of droplets, which represent the dried remnants of droplets and may contain infectious pathogens [2].

Several studies on the number and size of the saliva droplets and other secretions from the respiratory tract have been published [3,4]. These studies and reviews indicate that the size of the droplet cores due to sneezing, coughing, and speaking will probably be the function of the generation process and environmental conditions. The real distribution of the droplet size also depends on parameters such as, for example, the exhaled air speed, liquid viscosity, and flow course (by nose, mouth, or both). The infectious aerosol is the set of particles in the air charged with pathogens. The aerosol particles may be deposited in a sensitive individual by inhalation. The aerosol transfer is biologically acceptable when infectious aerosols are derived from the infected person [5].

Currently, humankind is endangered by the pandemic of a hard acute respiratory syndrome virus (SARS-CoV-2), which induces COVID-19 disease. It is a highly infectious and pathogenic virus infection which appeared, according to the current research, in the Chinese city of Wuhan [6]. According to Verity et al. (2020) [7], coronavirus represents a large family of pathogens, most of which cause mild infections of respiratory tract, such as, for example, common cold. However, the coronaviruses may also be lethal. An example of a dangerous coronavirus is SARS (Severe Acute Respiratory Syndrome) or COVID-19 (coronavirus disease 2019). This disease is spread between humans, by droplets from the respiratory tract of the infected person (through nose and mouth), especially during sneezing and coughing. A healthy individual can inhale these droplets or can bring them onto his or her face (to the eye mucous membrane, mouth, or nose) after the contact with the surface on which the virus is located. Scientists intensively study the transfer of COVID-19 and one of the main routes of infection transfer is droplet infection, when the virus particles spread through the air and then fall on the surfaces [8,9,10].

The qualitative analysis of comparing the transfer of COVID-19 and factors concerning its transfer in China, Korea, Japan, Italy, USA, and Brazil were the object of an earlier study [11]. Another example of the transfer view is documented in the study of Liu [12], who indicated the model and analysis of factors influencing the transfer of COVID-19 in the municipal environment between the municipalities. In addition, there are increasingly more studies of epidemiologic differences between municipal and country regions [13,14]. The pandemic caused by SARS-CoV-2 is of critical concern worldwide. Many places, especially large cities in Europe and the USA, are seriously impacted [15,16]. After the COVID-19 breakout, scientists remain active and focus special attention to this epidemic. According to Xu et al. [17], the development of the new coronavirus may be very complicated (but also despite this fact, increasingly more scientists will devote their attention to this issue). An example of this is also the study from the environment of the Czech Republic [18] or at the forest in Slovakia [19]. In relation to the quality of life and environment, this phenomenon is analyzed in [20]. According to Sannigrahi et al. [21], it is very important to point out the relationship between mortality rate and the sociodemographic composition of the population. The authors [22,23,24] also analyze the impact of COVID-19 on teaching. Rose-Redwood et al. [25] point out that the COVID-19 pandemic has a completely different spatial character, and the value of geographic theory and practice provide critical (what is happening) and normative (what should be happening) thinking, in addition to applied results (performance of facts). The studies in terms of time and space patterns of the COVID-19 pandemic in the regional scale of Europe and its countries are analyzed in [26]. In the context of improved air quality due to COVID-19 in cities worldwide, this phenomenon is being investigated [27]. COVID-19 had a negative impact on the economies of individual countries. For example, Slovakia’s gross domestic product fell by 5.2 percent in 2020. The first wave of the COVID-19 pandemic caused historic declines in key indicators in the second quarter. However, the economy managed to start at the end of the year despite the second wave of the pandemic. Growing foreign demand had a decisive portion in the result [28]. As most of the published studies focus on Asia and America, there is a need for comparably more spatial studies using geographically detailed data in other regions of the world [29]. COVID-19 is one of the infectious respiratory diseases and its level of risk was modeled in Slovakia through selected indicators.

## 2. Materials and Methods

The spatial divergence of COVID-19 disease on a global level and its increase and growth differences regionally in individual countries currently represent important problems. Slovakia is one these countries. A typical example of regional disparities from the point of view of COVID-19 is the so-called COVID automat that depicts the actual real status in individual regions of Slovakia. The primary condition on which the successful solution for lowering regional disparities of COVID-19 in Slovakia depends significantly on the analysis at local levels. That is why our observation units of risk factor monitoring focused on the municipalities of Slovakia as the smallest administrative units. For the smaller spatial units, there are no consistent data sources, which is why risk level calculations, for example, are not possible for areas of 1 × 1 km.

The difficulty of detailed analysis of the monitored phenomenon of risk rate for municipalities in terms of COVID-19 disease and the capability of mathematical and statistical methods indicate that the simplest methods for measurement and evaluation of regional disparities are indirect methods based on the point method. The basic advantage of these methods is their transparency of indicator completion, the potential for comparing data with results, and their capability for combining the indicators into one synthetic characteristic. In our case, this synthetic characteristic creates a qualitative risk map. The advantage of the point method is its ability to combine the indicators in various units into one synthetic characteristic that exhibits a single dimension. Its disadvantage is that the point method is based on the absolute variability of indicators while unable to reach their relative variability [30]. The environment and its characteristics, together with preventive measures, influence the rate of impacts of special events. According to Hadi and Mahrokh [31], the environmental factors that are important in the transmission of SARS-CoV-2 are very important. For example, the maximum relative humidity, maximum temperature, and maximum wind speed in four cities in China and five cities in Italy were processed in a study on the effect of these factors on COVID-19. This fact is also analyzed in other studies [32,33,34]. Vulnerability may be considered as one of the environmental characteristics that directly describe the ability of the environment to resist the risk or special event [35]. We evaluated the analysis of the overall risk assessment of Slovak municipalities with COVID-19 disease through selected population indicators of Slovakia. We placed emphasis on the local level. These are variables that entered into the overall synthesis. Subsequently, this phenomenon was shown through map output of the relative risk in Slovak municipalities. The variables entering the synthesis acquire only the socioeconomic character of the population.

Currently, the notion of vulnerability is used in all the domains of safety, from information safety to the environment, but also in psychology, economy, and geography, among others [36]. According to Galinna et al. [37], vulnerability is part of the definition of risk, and it functions as the degree of endangerment.

R = H × V, where R is risk, H is hazard, and V is vulnerability.

According to Cardona et al. [38], the period of exposure to a given risk enters into the relationship. According to [38], the risk is not a function of hazard, vulnerability, or exposure to endangerment [36].

R = H × E × V, where R is risk, H is hazard, E is exposure.

Within the analysis of vulnerability of the affected territory, the following indicators enter our evaluation: age of the citizens, access to water, gypsy villages, social service centers, percentage share of deaths in the municipalities, and selected diagnoses. The age composition of the citizens is one of the important risk factors from the point of view of spreading COVID-19 disease because the most endangered group of citizens are people of greater age. That is why we used an age limit of 60 years and we monitored more closely its percentage representation in the individual municipalities. The access to water is the next factor with a significant impact on the transfer of the disease, which was analyzed via indicators of connection among municipalities to toilets and showers. The vulnerability of the territory also included the presence of gypsy villages as a factor. Within this marginalized group, we monitored the number of people in the concentration, the share of the gypsy citizens from the total number, and access to water—water duct or well. Due to the fact that COVID-19 especially endangers the people of greater age, we considered it also important to include in the vulnerability of the territory an indicator of the presence of social service centers. We focused our attention on the number of the social service centers together with their capacity. For each municipality, the mortality on cardiovascular and respiratory diseases was also evaluated. We determined the total vulnerability by totaling the individual partial factors and its proportional division into the resulting qualitative vulnerability classes.

For exposure, which is defined as the risk exposure of the citizens, we used the indices of assembling and remoteness as the data input sources for its evaluation. For the index of assembling, we focused our attention on the objects that have the greatest risk, based on the point of view of risk of disease transfer between citizens. For each municipality, the number of individual objects was defined (cemeteries, churches, shops, parks, filling stations, large train stations, and bus terminals), which was recounted and classified into several classes. In order to define the index of remoteness, we used the following indicators: density of citizens in the town residential areas, distance from traffic routes, and accessibility of public transport. The density of citizens in the town residential areas was calculated on the basis of residential area of the municipality and its number of citizens. The rate of exposure of the individual municipalities, which defines to what extent the inhabitants may be exposed to the infection, together with the rate of their vulnerability, which treats the supposed consequences in case of the infection breakout, were the combined source data for defining the complex risk level for the individual municipalities. In other words, if the citizens of the municipality are highly vulnerable and at the same time there is a high level of exposure to the potential infection, then the municipalities are at high risk. The empirical results are displayed with the focus on the marginal effects of the key variables. Based on the character of the available data and the applied analytical approaches, the contribution may be classified from the methodological point of view as one of the analytical studies. These are typical especially for a spatial domain in epidemiological research practice. The basic database for the data processing and analysis was provided by the statistics office of the Slovak Republic.

## 3. Results

### COVID-19 in Slovakia

The first case of COVID-19 was recorded in Slovakia on 6 March 2020, less than four months after its discovery in China. According to the Public Health Authority of the Slovak Republic, the disease was imported to Slovakia from 52 countries, mostly from Austria, United Kingdom, and Germany. Due to the endangerment of public health, the government of the Slovak Republic announced on 11 March 2020 an emergency situation on its territory. On the 12 March 2020, the Central Crisis Staff of the Slovak Republic adopted several measures in connection with COVID-19. Starting on 13 March 2020, a 14-day home quarantine became obligatory, all three international airports were closed, and international and national train and bus transport was limited. Several business sites were closed: natural and artificial swimming pools, sporting facilities, and facilities for the children and the young. Public catering services were closed (confectioner shops, cafeterias, bars, and similar public catering facilities), except for the restaurants and fast food facilities. At the same time, operation of wellness centers and entertainment centers such as casinos, cinemas, and other leisure time activities, e.g., ski centers, were forbidden. Organizing mass events of sporting, cultural, social, or other character was forbidden. Visiting hospitals and all public and nonpublic social service providers was also forbidden. On 16 March 2020, all the preschool and school facilities were closed. By these measures, Slovakia tried to eliminate the spreading virus. Despite these measures, it was necessary to focus attention, in terms of further spreading of the disease, on the social and economic phenomena at the local level.

**Figure 1:**
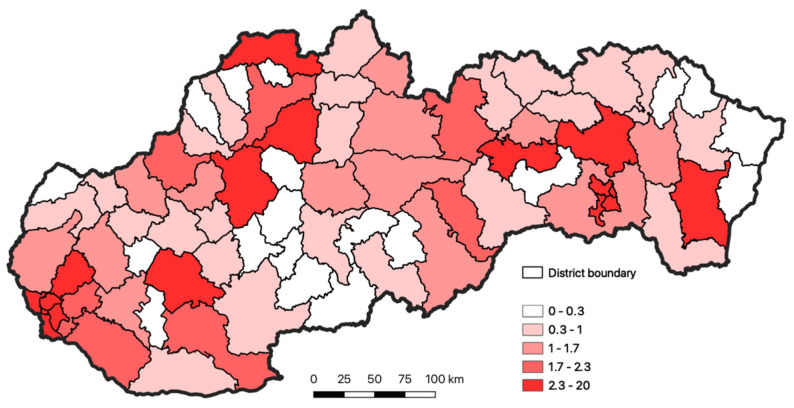
Occurrence of COVID-19 disease in the individual districts of Slovakia for the period March–July 2020 (the % share of occurrence of the disease in the district from the total number of disease in Slovakia). Source: https://github.com/Institut-Zdravotnych-Analyz/covid19-data, accessed on 30 October 2020.

**Figure 2:**
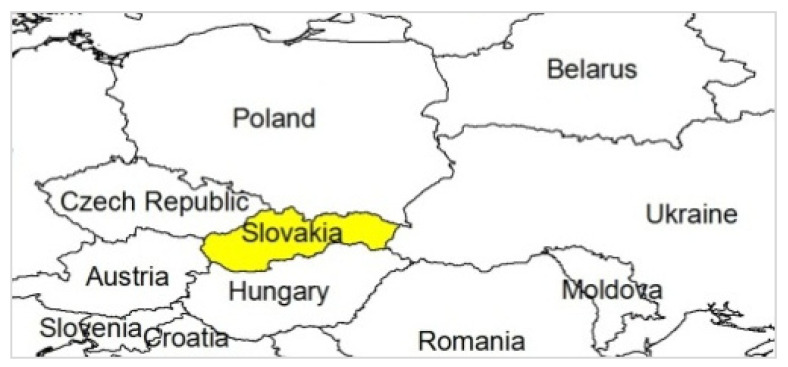
Location of Slovakia.

**Figure 3:**
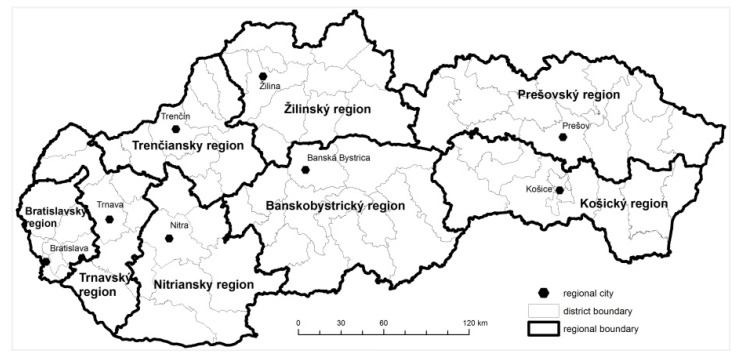
Regional divisions of Slovakia.

Strict rules and measures, which were adopted at the beginning of the pandemic, helped to keep the number of the infected persons during spring and summer under control. When the measures were gradually released, the number of the cases began to rise. From the point of view of the spreading disease, it was very important to focus attention on the regions and districts where the virus was the most widespread. The occurrence of the disease on the district level of Slovakia is documented in Figure 1. For the entire period monitored, from the 6 March 2020 to the 31 July 2020, the total number of infected persons in Slovakia was 2337. The location and regional division of Slovakia is shown in Figure 2 and Figure 3.

**Figure 4:**
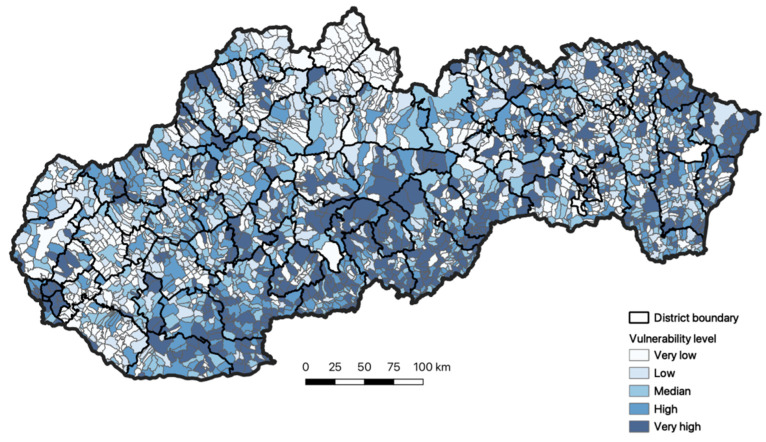
Vulnerability of the inhabitants of Slovakia toward respiration diseases. Source: Statistical Office of the Slovak Republic, 2020.

Figure 4 documents the spatial differentiation of vulnerability of Slovakia’s inhabitants. We can see the spatial arrangement of the individual vulnerability levels locally, which are classified into five categories. From the point of view of the phenomenon monitored, we can point out in Slovakia the regions with very high vulnerability. One region includes the municipalities of northeastern and the easternmost part of Slovakia. These are the municipalities in the Prešovský region, especially. In the southern part of Slovakia, particularly in the territory of the Banskobystrický region and Nitriansky region, there is a domain with a very high level of vulnerability. In addition to these homogenous regions, very high vulnerability also occurs locally in other regions of Slovakia. In some of the regions, we recorded the presence of the local focal points, which are very often augmented by a very poor economic situation in the region. The absence of the employment forces the inhabitants of the region to travel for work out of Slovakia. Their repatriation from Great Britain and Germany, for example, but also the neighboring Czech Republic, represents a very high endangerment for the domestic inhabitants.

**Figure 5:**
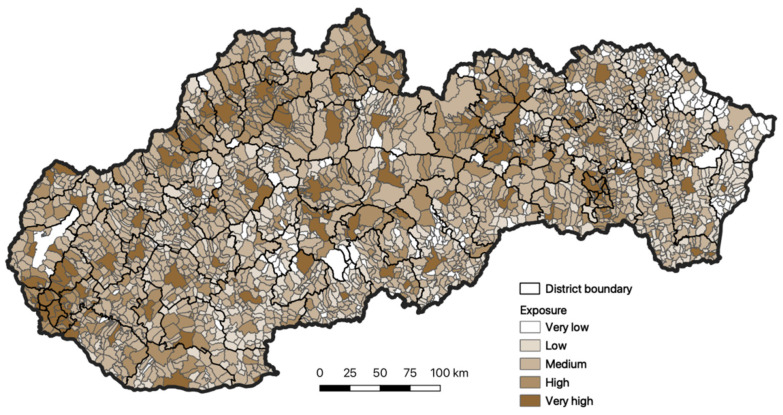
Exposure of inhabitants of Slovakia to respiratory diseases. Source: Statistical Office of the Slovak Republic, 2020.

Based on a more detailed analysis of the spatial distribution of exposure of the inhabitants, it is also obvious that in Slovakia there are municipalities that are classified in the very low or middle exposure categories (Figure 5). We identified very high exposure in the domain of western Slovakia with dominant representation in the Bratislavský region. This category is also locally visible in other regions of Slovakia.

**Figure 6:**
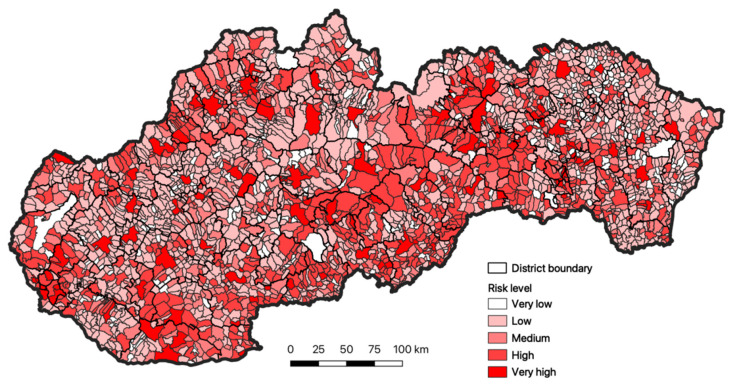
The total evaluation of the risk for the inhabitants of Slovakia to respiratory diseases. Source: Statistical Office of the Slovak Republic, 2020.

The total evaluation of risk in the municipalities of Slovakia is the synthesis of the two previous parameter categories (vulnerability and exposure), based on the point of view of individual risk levels. We may state that the easternmost regions of Slovakia reach very low or low risk level. One is the domain of the Prešovský and Košický region. Toward the west lies the category of the middle or high risk level. Within the territory of Slovakia, we also identified municipalities that are categorized with very high risk level (Figure 6).

**Figure 7:**
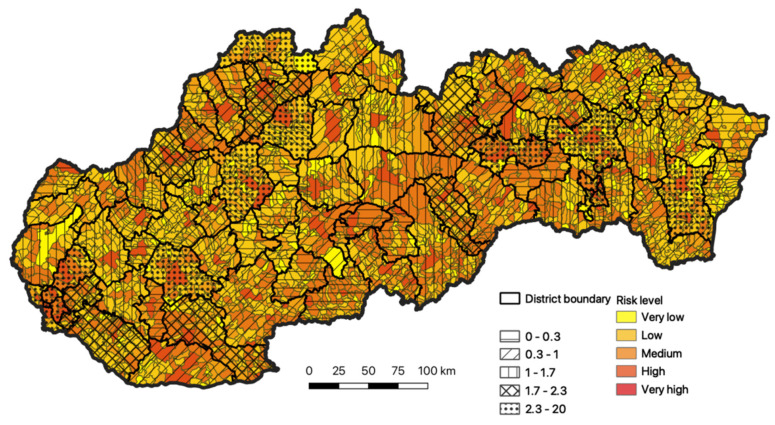
Comparison of the total evaluation of risk with the actual recorded status, as of 31 July 2020. Source: Statistical Office of the Slovak Republic, 2020.

In the final phase of research, the resulting total evaluation of the risk for the inhabitants of Slovakia, documented in Figure 7, was compared to the total occurrence of the diseases in Slovakia, depicted in Figure 2.

Our modeling confirmed the regions with the highest intensity, especially in the districts of Bratislava and Košice, and also in the regions of Nitra, Prešov, Michalovce, Spišská Nová Ves, Prievidza, Martin, and Čadca. The second highest intensity of concentration is recorded in the districts of western Slovakia (Nové Zámky and Dunajská Streda), toward the north these are the districts of Trenčín and Žilina. This group is complemented by the districts of Poprad and Revúca.

We compared our model with the first wave of COVID-19 propagation. The next wave was influenced by external factors. In spite of the fact that other factors affect the phenomenon monitored, modeling confirmed a relatively high precision and we were able to predict the domains of Slovakia characterized by the greatest risk.

## 4. Discussion

The world is coming through a pandemic whose consequences are incomparable to anything we have known for the last century. The life in the world, as well as in Slovakia, slowed for some time due to a small microelement that we cannot eliminate and not easily defined. One of the important aspects concerning the spread of this disease is globalization, which has both positive impacts and concomitant risks.

The influenza epidemics/pandemics have been and continue to be a part of the life of the population. Under the current situation, however, it is impossible to predict when, where, and with what force they will occur again. The COVID-19 breakout, which was declared to be a global pandemic, continues to overload the healthcare system by increasing numbers of patients with clinical symptoms of coronavirus. This increasing level and occurrence of infection presents its own challenges, which overloads healthcare institutions, the global economy, and affects the physical and mental health of people worldwide. Even if several strategies to lower the consequences of this virus disease are adopted, rural inhabitants may not be sufficiently considered, even despite a high risk of morbidity and mortality within this population group [39].

In the beginning of the pandemic, the strategy of the immediate and correct reaction to the efficient slowing of the COVID-19 was very important. The town domain and density of the inhabitants were negatively related to the viral spread in the first phase of the epidemic [12]. As many biological and physiological characteristics of this new disease remain unknown, the statistical analyses specified in this document may contain valuable information. The key elements for the spread and consequences of the actual pandemic, according to several studies, may include the demographic structure of the inhabitants and the level of intergeneration social contacts [40,41]. The current COVID-19 pandemic is a strong reminder of the fact that urbanization has changed the way people and communities live, work, and interact. That is why it is necessary to strengthen the systems and local capacities in order to prevent the spread of infectious diseases [14]. One of the important indicators playing an important role at the infection is the relation of DNA content with resistance toward coronavirus. SARS-CoV-2 attacks the cells in a manner similar to that in HIV, using the furine enzyme, the same as Ebola and HIV, because this virus received the so-called HIV-like mutation. About 1% of northern European inhabitants, especially Swedish, are immune to HIV infection. Another 10–18% of Europeans are at least partially immune toward the disease, which represents a lowering of the infection risk; if they do get infected, they experience a slow progression of the disease [42]. If there is a natural genetic resistance toward AIDS, however, it is highly probable that there is also the natural resistance towards COVID-19. A certain degree of resistance towards HIV in a part of the population is a proven fact. Apparently, there is a similarity for the SARS-CoV-2 infection. This highly probable resistance toward COVID-19 within a large part of the European population is certainly not connected with the male Y chromosome, thus men would not have more than double mortality than women. The male haplo groups indicate only a certain genetic relation/difference of the individual ethnics. Of course, this is not the resistance toward COVID-19 only in the Slavic DNA R1a, but also other ethnic groups, for example, Norse DNA [43]. For us, our DNA is important.

It is highly probable that COVID-19 was here much earlier than when we recorded it for the first time. We considered those severe symptoms, however, as a consequence of influenza. Even if, in the reciprocal view, many patients indicated all the typical signs of coronavirus infection, tests were not done at that time [44]. The genetic specification of the nations differs much more. The empirical data specified in this document may also have political consequences. As the epidemical situation still continues and is quickly developing and changing in worldwide, the present study also provides a timely analysis of the spreading infection within the territory of Slovakia.

## 5. Conclusions

This study proved a high importance of studying how an emergency event in the public health domain may influence the life of the inhabitants. This article focused on predicting and identifying the potential rate of endangerment in Slovakia. Our analysis confirmed the correlation between the status of people infected, identified at the beginning of the pandemic in Slovakia, and the total COVID-19 risk level. The result reached was confirmed on the basis of our selected indicators by spreading, and this fact was reflected also in the first wave. The spread and transfer of the disease was influenced much more by the exposure presented, especially regarding traffic availability, but also by the concentration of inhabitants in the locations selected (shops, cemeteries, and others). We managed to predict with a very high precision the most risky regions of Slovakia. The prediction model developed may be also used, implementing the indicators provided, for the conditions of other countries in central Europe. Our contribution should be the source for further research on the issue. Trying to develop better prediction models in the future, we also propose to include other variables that reflect the total level of the individual regions in time. The medical and geographical procedures will play decisive role during the current pandemic as well as after the end of this crisis. The use of the risk evaluation concept may provide valuable information not only on the risks, but also on risky localities, thus enabling the inclusion of measures to lower the risk of an emergency event occurrence, as seen in case of COVID-19.

It is not the modeling of the spreading infection by which it would be possible to predict the global pandemic spreading. The municipalities risk map may be the tool to lower the consequential impacts in the municipality itself. The municipalities with high risk require more precise maintenance of the measures, more strict limitations of movement, and stricter tracing of the potential disease occurrence. In contrast, in municipalities with lower risk it is possible to adopt less strict measures. We are aware of the fact that the analyses of vulnerability and assembling should also include, for example, other factors such as the number of inhabitants going to work in another district, the number of employees going to work in the neighboring countries at the borders, and the number of diagnosed diseases except for the number of the deaths. These data are not accessible, however, and that is why they are not included in the analysis. It is also important to realize the dynamics of the disease development and the new mutations that may occur. Conversely, there is a rising percentage of vaccinated highly vulnerable people, which lowers the importance of the criteria we used, for example, social service centers and the number of the inhabitants over 60 years. Just the same as many other phenomena, the COVID-19 pandemic showed us its own geography.

## Data Availability

The data presented in this study are available on request from the corresponding author. The data are not publicly available due to privacy reasons.
